# Soil-Associated Bacillus Species: A Reservoir of Bioactive Compounds with Potential Therapeutic Activity against Human Pathogens

**DOI:** 10.3390/microorganisms9061131

**Published:** 2021-05-24

**Authors:** Galal Yahya, Asmaa Ebada, Eman M. Khalaf, Basem Mansour, Nehal A. Nouh, Rasha A. Mosbah, Sameh Saber, Mahmoud Moustafa, Sally Negm, Mohamed M. A. El-Sokkary, Ahmed M. El-Baz

**Affiliations:** 1 Department of Microbiology and Immunology, Faculty of Pharmacy, Zagazig University, Al Sharqia 44519, Egypt; 2 Department of Microbiology and Biotechnology, Faculty of Pharmacy, Delta University for Science and Technology, Gamasa 11152, Egypt; asmaaebada89@yahoo.com (A.E.); Elbaz_pharmacy@yahoo.com (A.M.E.-B.); 3 Department of Microbiology and Immunology, Faculty of Pharmacy, Damanhour University, Damanhour 22511, Egypt; eimanpharmacist@gmail.com; 4 Department of Pharmaceutical Chemistry, Faculty of Pharmacy, Delta University for Science and Technology, Gamasa 11152, Egypt; basem2412@yahoo.com; 5 Department of Microbiology, Albatterjee Medical College, Jeddah 6231, Saudi Arabia; nehalahmed_nouh@yahoo.co.uk; 6 Infection Control Unit, Zagazig University Hospitals, Zagazig University, Zagazig 44519, Egypt; rashamosbah6@gmail.com; 7 Department of Pharmacology, Faculty of Pharmacy, Delta University for Science and Technology, Gamasa 11152, Egypt; sampharm81@gmail.com; 8 Department of Biology, College of Science, King Khalid University, Abha 9004, Saudi Arabia; mfmostfa@kku.edu.sa; 9 Department of Botany and Microbiology, Faculty of Science, South Valley University, Qena 83523, Egypt; 10 Life Sciences Department, College of Science and Literature Mahyel Aseer, King Khalid University, Abha 61413, Saudi Arabia; snsir@kku.edu.sa; 11 Unit of Food Bacteriology, Central Laboratory of Food Hygiene, Ministry of Health, Sharkia 44516, Egypt; 12 Microbiology Department, Faculty of Pharmacy, Mansoura University, Mansoura 35516, Egypt; m_elsokkary2022@yahoo.com

**Keywords:** soil bacteria, *Bacillus subtilis*, antibiotics, MRSA, glycopeptides

## Abstract

Soil hosts myriads of living organisms with the extensive potential to produce bioactive compounds. Bacteria are the major soil inhabitants that represent a rich reservoir for antibiotic production along with their role in recycling nutrients and maintenance of the soil ecosystem. Here, from 55 tested soil samples, we isolated and identified a novel antibiotic-producing bacterial strain with a phylogenetically closest match to *Bacillus subtilis* sp. based on BLASTN search of GenBank for the 16S rRNA gene sequence. We characterized this novel strain through microscopic, biochemical, and molecular techniques, combined with testing its potential antimicrobial activity. Chemical studies revealed that the antibiotic produced by this strain is a glycopeptide. It exhibited profound activity against both methicillin-resistant *Staphylococcus aureus* (MRSA) and *Candida albicans*. The antibiotic is optimally produced at 37 °C after 28 h of growth. The biocompatibility of the extracted antibiotic was tested over a wide range of factors including temperature, pH, surfactants, and metal salts. To confirm its therapeutic potential, a sterile solution of the antibiotic was tested in vivo against bacteria-induced keratitis in rats where significant healing activity was recorded. Hence, this soil Bacillus strain may lead to the development of novel antibiotics for the treatment of human pathogens.

## 1. Introduction

Bacteria are the most plentiful microbial communities in the soil, where they play fundamental roles in the maintenance of the soil ecosystem, recycling soil nutrients, mineral enrichment, nitrogen fixation, and improving the soil biogeochemical properties, which is necessary for crop production [1,2]. In addition, many antibiotic-producing microorganisms could also be isolated from soil [3,4]. Antibiotics are one of the secondary metabolites produced by microorganisms and represent the first choice usually selected to manage microbial infections of bacterial origin. In the same respect, most of the widely used antibiotics are produced from a limited genera of microorganisms, mainly *Penicillium* sp., *Streptomyces* sp., *Cephalosporium* sp., and *Bacillus* sp. [5]. These antibiotic-producing microorganisms are widely studied to identify new forms of antimicrobial compounds as alternatives to combat emerging multidrug-resistant microorganisms.

Soil bacteria represent the primary source of polypeptide antibiotics that are used against various pathogens, particularly those that are drug resistant [6]. *Bacillus* is the major bacterial genus that exists in soil, and it produces many valuable antibiotics [7]. Antibiotics extracted from *Bacillus* sp. have been tested to define their antimicrobial spectrum. Radical differences exist among the antimicrobial compounds produced by *Bacillus*, as some exhibit a narrow spectrum of activity, whereas others have a broad spectrum [8,9,10]. Antibiotics with efficacy against multidrug-resistant bacteria are of special importance [11,12]. *Bacillus subtilis* is a major bacterial species that produces effective antimicrobial compounds against multidrug-resistant pathogens such as methicillin-resistant *Staphylococcus aureus* (MRSA) [13]. MRSA is the main pathogen that causes nosocomial infections resulting in bacteremia associated with high morbidity and mortality rates [14]. The danger of MRSA is not only because of its being a frequent cause of hospital infections but also because of its increasing ability to acquire resistance to various antibiotics, including ß-lactam, aminoglycosides, fluoroquinolones, clindamycin, chloramphenicol, and tetracycline [15,16,17,18,19,20]. Thus, there is an urgent need to discover and develop new antibiotics because of the increasing frequency of MRSA resistance to vancomycin and teicoplanin, which are the final therapeutic options against this pathogen [7,21,22,23,24].

In the present study, we identified an antibiotic-producing bacterium from a screen of bacterial species isolated from soil samples by sequencing the 16S ribosomal DNA gene. The isolated bacterium was found to belong to the genus *Bacillus.* We further investigated the optimal growth conditions for antibiotic production, stability in the presence of various metals, and its growth over a wide range of temperatures and pH levels. Furthermore, we examined the chemical identity of the antibiotic and evaluated its antimicrobial activity in vitro and in vivo.

## 2. Materials and Methods

### 2.1. Soil Sample Collections and Isolation of Bacterial Isolates

Fifty-five soil samples were collected from different environmental areas in Mansoura city, Egypt. Firstly, the soil samples were dried by spreading them on filter paper and leaving them at room temperature for 2 days (48 h) where a dry conditioner on dry mode was used, and then any plant fragments or rocks were removed by using a sieve (or by sieving) [25]. After drying, 1 g of each soil sample was suspended in 10 mL of saline (0.85%) and processed by heating at 60 °C for 60 min in a water bath. The saline was serially diluted 10-fold and cultured in nutrient broth (NB) followed by culturing on nutrient agar for 24–48 h at 37 °C [26]. Each morphologically unique bacterial colony was picked up and streaked on a fresh nutrient agar plate for further purification and then cultured in LB broth (10 g l^−1^ tryptone, 5 g l^−1^ yeast extract, and 5 g l^−1^ NaCl, pH 7.2) to prepare 50% glycerol stocks stored at −80 °C. The bacterial strains were fully characterized by Gram staining along with a series of routine biochemical tests. The genomic DNA for the isolates was extracted for molecular characterization as described previously [27]. The purified DNA was stored at −80 °C.

### 2.2. Biochemical Characterization of Bacterial Isolates

The bacterial isolates were tested, using the spot agar method, for the following enzymatic activities: amylolytic activity (starch hydrolysis), esterase activity, proteolytic activity (protease and gelatinase), and hemolytic activity. All assays were carried out using basal mineral salts medium (BMSM) with a specific substrate and in triplicate [28].

#### 2.2.1. Determination of the Amylolytic Activity

Each overnight bacterial culture (10 µL) was spotted on the surface of BMSM containing 0.01% soluble starch, with pH 6.0. The plates were incubated at 37 °C for 2 days. After incubation, the plates were treated with Lugol’s iodine solution, and the existence of clear halos around the colonies indicated amylase activity [29].

#### 2.2.2. Determination of the Esterase Activity

BMSM supplemented with 0.1 g l^−1^ CaCl2 and 0.1 g l^−1^ phenol red was prepared, sterilized, and supplemented with previously sterilized Tween 80 at a final concentration of 1% (*v*/*v*). Bacterial isolates were spotted and incubated at 37 °C for 2 days. The presence of clear zones around the colonies indicated positive esterase activity [28].

#### 2.2.3. Determination of the Proteolytic Activity

To check casein hydrolysis, skim milk (10 g L^−1^) was mixed with autoclaved BMSM at 45 °C and plated. The bacterial isolates were inoculated. Then, after a growth period, 2.0 mL of 0.1 mol/L HCl was added to the plates, and the presence of clear halos around the colonies was evaluated. Gelatin hydrolysis was determined using BMSM containing 4.0 g L/L bacteriological gelatin. After incubation at 30 °C for 24–48 h, the plates were covered with Frazier’s revealers (100 mL distilled water, 20.0 mL HCl, and 15.0 g mercury dichloride) [30]. The presence of a clear transparent halo around the bacterial colony determined proteolytic activity.

#### 2.2.4. Determination of the Hemolytic Activity

The tested bacterial isolates were spotted on the surface of blood agar plates and incubated overnight at 37 °C. The pattern of hemolysis was assessed where the bacteria were spotted.

### 2.3. PCR Amplification and Sequencing

The produced colonies were identified by sequencing the 16S rRNA gene. Samples were amplified in a 25 μL reaction mixture containing 2.5 μL DNA, 12.5 μL My Taq Red Mix (Bioline Co., London, UK), 1 μL of each primer (Table 1), and nuclease-free water to a final volume of 25 μL. Thermocycling conditions were as follows: an initial heating step at 94 °C for 5–10 min, 35 cycles of 94 °C for 30 s, annealing at 52 °C for 30 s, elongation at 72 °C for 45 s, and a final extension step at 72 °C for 3–5 min. The PCR products were separated on a 1.5% agarose gel along with the GeneRuler 100 bp Plus DNA Ladder or O’GeneRuler DNA Ladder Mix 10 kb (Thermo Scientific, Waltham, MA, USA), stained with ethidium bromide, visualized on a UV transilluminator, and photographed. PCR amplicons containing the 16S rRNA gene were cleaned up using the QIAquick PCR Purification Kit from Qiagen^®^, according to the manufacturer’s instructions. The DNA was eluted with Qiagen^®^ elution buffer, the concentration of DNA was measured by using a NanoDrop instrument (OPTIZEN NanoQ, Mecasys), and the purified PCR fragments were prepared for sequencing. For the latter, 5 μL of template DNA (20–80 ng for PCR samples) was mixed with 5 μL of the 16S rRNA gene-specific primers (5 pmol/μL). Barcoded PCR samples were sequenced on the Illumina HiSeq platform using 300 PE chemistry (GATC-Biotech, Konstanz, Germany, now part of Eurofins Genomics Germany GmbH).

### 2.4. Antimicrobial Susceptibility

The diffusion method based on the Clinical and Laboratory Standard Institute (CLSI, 2020) was used. Susceptibility testing was performed using Cefoxitin (FOX, 30 µg) to assign the MRSA strains among the collected clinical isolates of *Staphylococcus aureus*. The optical density of the bacterial suspension was adjusted to the turbidity of 0.5 McFarland turbidity standards and then streaked over a Mueller–Hinton agar plate using a cotton swab. Antibiotic discs were obtained from Oxoid, Hampshire, England. The results were interpreted according to the criteria indicated in the CLSI guidelines.

### 2.5. Determination of the Antimicrobial Producing Activity

#### 2.5.1. Media and Culture Conditions

All soil bacterial isolates were cultivated in tryptone soya broth medium (TSB) at 37 °C for 48 h. Various media including Luria–Bertani broth (LB), NB, Mueller–Hinton broth (MH), brain heart infusion broth (BHI), and TSB were used for culturing. The production of the antimicrobial molecule was maximal in TSB. The *Candida albicans* strain was grown on Sabouraud dextrose agar (SDA) [7]. All media were purchased from Oxoid, UK, and Hi-Media, India.

#### 2.5.2. Screening for Antimicrobial Activity

The spectrum of antimicrobial activity was tested against different indicator bacterial strains of reference and clinical isolates including Gram-positive cocci and Gram-negative bacilli, as well as pathogenic fungi obtained from the culture collection of Microbiology and Biotechnology Lab, Faculty of Pharmacy, Delta University (Table 2), using cut-well agar diffusion [13]; we selected this group of bacterial species because they are commonly involved in different infections of major clinical challenge on the public health in our environment in particular and due to dramatic increase in antibiotic resistance elaborated by these pathogens in general. The bacterial strain, *S. aureus* ATCC 29213, was initially used as an indicator for screening purposes against Gram-positive cocci; then, our screening was extended against clinical isolates of MRSA. Briefly, each bacterial isolate was grown in 100 mL of TSB at 37 °C for 2 days with constant shaking at 150 rpm. The cells were harvested by centrifugation at 12,000 rpm for 20 min at 4 °C, and the supernatant was collected and filtered through a syringe filter (0.45 μm pore size). Next, 100 µL of the indicator organism was spread on MH agar plates, and wells of 6 mm were prepared with a sterile cork borer. Supernatants (100 µL) were added into the wells representing each indicator strain, and the zone of inhibition was measured in mm after incubating for 2 days [33].

#### 2.5.3. Effect of Medium Type for Optimum Antimicrobial Activity Production

Various broth media were tested to detect the best culture medium for optimal antimicrobial productivity, including tryptic soy broth (TSB), Luria–Bertani broth (LB), nutrient broth (NB), Mueller–Hinton broth (MH), and brain heart infusion broth (BHI). The pH of the medium was adjusted to 7.0 before autoclaving (121 °C, 15 min). Bacterial culture (1 mL) was inoculated into 50 mL of each medium and incubated at 30 °C with shaking at 150 rpm for 2 days. After incubation, the supernatant was tested for the production of antimicrobial activity using a cut-well agar diffusion assay [7].

#### 2.5.4. Kinetic Production of Antimicrobial Activity

The bacterial isolate was inoculated into 100 mL of TSB and incubated at 37 °C while being shaken. Every 2 h a sample was aseptically collected and centrifuged at 15,000 rpm for 25 min. The supernatant was tested for antimicrobial activity against clinical MRSA isolates using the cut-well agar diffusion assay. Growth kinetics were monitored spectrophotometrically (GENWAY 6051, UK) at 600 nm (A600), and the activity was recorded by measuring the diameter of the inhibition zone after the cut-well diffusion assay [13].

### 2.6. Purification and Biocompatibility Studies of the Isolated Antimicrobial Molecule

#### 2.6.1. Partial Purification of the Antimicrobial Molecule

The *Bacillus* sp. strain was grown at 37 °C for 44 h in TSB, and the cell-free supernatant was collected by centrifugation at 12,000 rpm at 4 °C for 20 min. The supernatant was treated with 1N HCl to obtain a pH below 2 and stirred overnight in a cold room. The resulting precipitate was collected by centrifugation at 13,000 rpm at 4 °C for 25 min and dissolved in 20 mM sodium phosphate buffer, pH 8.0. The antimicrobial fractions were extracted by stirring with an equal volume of methanol (50% *v*/*v*) for 3 h and centrifuged to collect the supernatant. The methanol was evaporated completely at 55 °C, and the residue was dissolved in chloroform. Adsorption chromatography was performed using silica gel (230–400 mesh) equilibrated with chloroform [13].

#### 2.6.2. Effect of pH and Temperature

The effect of pH on the stability of the antimicrobial molecule was determined by adjusting the supernatant pH from 2.0 to 10.0 with 1N HCl and 1N NaOH and incubating at 37 °C for 2 h, neutralizing to pH 7, and testing against clinical isolates of MRSA1. The effect of the temperature was determined by incubating aliquots of the supernatant at different temperatures ranging from 40 to 100 °C for 30 min and also after autoclaving at 121 °C for 20 min. After cooling at room temperature, the antimicrobial activity was determined, and the untreated supernatant was used as a control [34].

#### 2.6.3. Effect of Surfactants

The effect of surfactant was determined by incubating the supernatant at 37 °C for 5 h with sodium dodecyl sulfate (SDS) and Tween 80 at a final concentration of 1% (*v*/*v*). Surfactants were prepared as a 10% stock solution in MilliQ water and sterilized through a 0.45 μm filter. The untreated sample and the surfactants at a final concentration were used as positive and negative controls, respectively [35].

#### 2.6.4. Effect of Metal Salts

The effect of metal salts on the stability of the antimicrobial molecule was evaluated by incubating the supernatant with metal salts (MgSO_4_, FeSO_4_, AgNO_3_, ZnSO_4_, CdCl_2_, CuSO_4_, NiCl_2_, and CaCl_2_) at a final concentration of 1 mg/mL at 37 °C for 1 h before testing for activity against the MRSA isolate. The untreated sample and the metal salts at final concentrations were used as positive and negative controls, respectively [36,37].

### 2.7. Chemical Characterization

#### 2.7.1. Ninhydrin Test: (Detection of Amino Acids)

The ninhydrin solution was prepared by dissolving 0.2 g of ninhydrin in 10 mL of ethanol and 1% of Bacillus extract in distilled water. Next, 1 mL of Bacillus extract solution was transferred into a dry test tube, and 1 mL of distilled water was added to another dry test tube as a negative control. Then, two to three drops of ninhydrin were added to each test tube, and both were gently warmed in a water bath (30–40 °C) for 5 min [38].

#### 2.7.2. Sudan III Test: (Detection of Lipid)

A stock solution of Sudan III was prepared by dissolving Sudan III in 99% isopropanol. 6 mL of this stock solution was taken and diluted with 4 mL of water and incubated for 5–10 min. The diluted stock solution was filtered and used directly for staining by adding three to five drops to both test tubes, one containing the 1 mL of Bacillus extract solution (tested sample) and the other containing 1 mL of distilled water (negative control). Both test tubes were shaken well and gently warmed [39].

#### 2.7.3. Molisch Test: (Detection of Carbohydrates)

The Molisch reagent was prepared by dissolving 0.5 g of α-naphthol in 10 mL of ethanol. In two dry, labeled test tubes 2 mL of Bacillus extract was transferred into one of them and 2 mL of distilled water was added to the other as a negative control. Next, two to three drops of Molisch reagent were added to each test tube. Finally, 1 mL of concentrated H2SO4 was gently pipetted along the side of each test tube [40].

### 2.8. In Vivo Study for the Antimicrobial Efficacy of Bacillus Extract

#### 2.8.1. Animals

Adult male Sprague-Dawley rats (250 ± 20 g) were obtained from the animal facility at the Faculty of Pharmacy, Delta University for Science and Technology (DU), Egypt. All animal care and experimental procedures were approved by the Institutional Animal Care and Use Committee at the DU and carried out in accordance with the relevant guidelines and regulations [41].

#### 2.8.2. Induction of Keratitis

After anesthetization using an intraperitoneal injection of ketamine (50 mg/kg)–xylazine (10 mg/kg) mixture [42], the cornea of the right eye of the immunocompromised rats was scarred using a 27-gauge needle. Ten microliters of 0.6% acetyl cysteine were pipetted onto the cornea to disintegrate the tear film and then flicked off using saline solution. A 10 µL suspension containing (5 × 108 CFU) of MRSA was applied to the scarred corneas. The left eye from each animal served as a control and was scarred using the same procedure but was not infected.

#### 2.8.3. Experimental Design

For the quick induction of keratitis, rats were immunocompromised with methyl prednisolone (50 mg/kg) for three successive days—before infection, the day of infection, and 1 day after infection. Rats were allocated into two groups as follows: untreated immunocompromised rats that were infected with MRSA (group A; *n* = 6) and immunocompromised rats that were infected with MRSA and treated with Bacillus subtilis extract (group B; *n* = 6) (Table 3). Administration was initiated 3 days after infection. The infection was defined by an ophthalmologist who was blinded to the protocol. A clear visual, gloomy appearance of the corneas was observed at the third day post-MRSA infection. Treatment was continued for an additional 2 days until the visual gloomy look of the corneas was entirely contracted in one animal from group B.

### 2.9. Statistical Analysis

Statistical analysis was performed using GraphPad Prism software version 8.0.2 (GraphPad Software Inc., La Jolla, CA, USA). Differences between groups were analyzed by an unpaired t test. Data are presented as the mean ± standard deviation (SD). A value of *p* ≤ 0.05 was considered statistically significant. For evaluating the antimicrobial activity at different pH levels and temperatures; experiments were performed in triplicates. Data are expressed as percentage of control value ± standard deviation. GraphPad Prism software version 8 (GraphPad Software Inc., La Jolla, CA, USA) was used to perform statistical analysis. Unpaired t test was used to assess the significance of difference between groups. * statistically significant at *p* < 0.05.

### 2.10. Phylogenetic Tree Construction

A nucleotide blast was performed within the *Bacillus* species (taxid:1386). The 50 most related organisms were used to calculate a phylogenetic tree based on the “fast minimum evolution” algorithm [43]; a maximum sequence difference of 0.75 was used, and the tree was exported as a .nex file with taxonomic names. The web app EvolView was used to visualize the distance tree [44].

## 3. Results

### 3.1. Strain Biochemical Characterization and Antimicrobial Spectrum 

**Figure 1:**
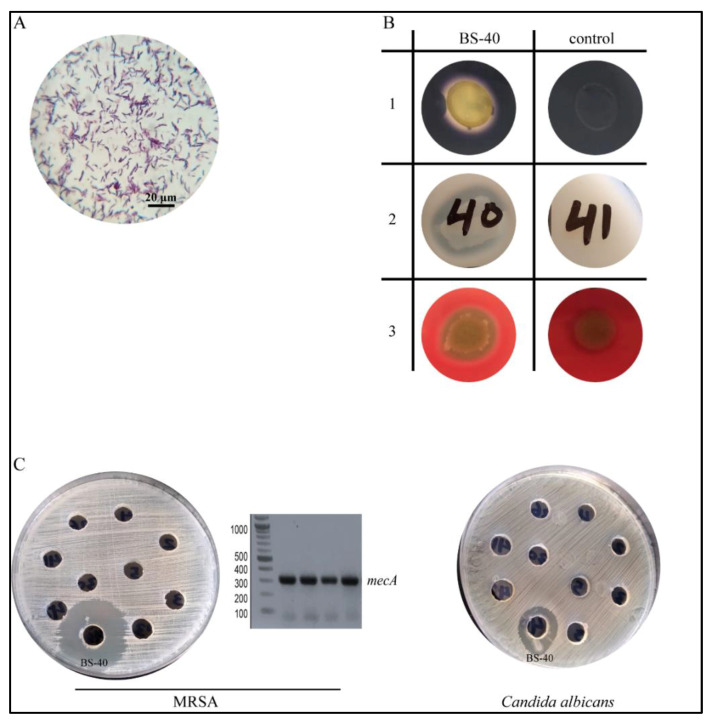
Screening for antimicrobial activity was carried out by well diffusion method and biochemical characterization of the antibiotic producing strains. (**A**) Gram stain film of antibiotic producing strain isolate BS-40, microscopic examination indicates Gram-positive rods (scale bar 20 µm). (**B**) Biochemical examination of isolate BS-40 for different enzymatic activities, such as amylolytic activity (1), proteolytic activity (2), and hemolytic activity (3); biochemical tests were performed according to standard procedures (see Materials and Methods section) and a control strain (non-amylolytic, non-proteolytic, and non-hemolytic) was plated in parallel for comparison. (**C**) Soil isolated bacterial strains were cultured, then supernatants were filtered, and each supernatant was filled into wells against the indicator strains (in this figure antimicrobial activity against MRSA clinical isolates and *Candida albicans*) and the zone of inhibition was measured in mm after 48 h of incubation (MRSA clinical isolates were identified by antibiotic sensitivity and existence of *mecA* gene tested by PCR).

Microscopic examination and Gram staining analysis identified Gram-positive bacilli (Figure 1A). A series of routine biochemical tests were performed to evaluate the enzymatic activity and hemolytic pattern of the isolated strain. The isolated strain was found to exhibit profound amylolytic activity with a 24 ± 4 mm halo zone, strong proteolytic activity when tested on skim milk agar showing a halo zone of 37 ± 6 mm, and enhanced gelatinase activity upon culturing on gelatin agar. No esterase activity was detected; however, a β-hemolysis growth pattern was observed by growth on blood agar (Figure 1B). The antimicrobial activity spectrum of the *Bacillus sp.* strain was evaluated by a cut-well agar diffusion assay. The supernatant prevented the growth of several Gram-positive bacteria species, including *Staphylococcus aureus*, *Staphylococcus epidermidis*, and various clinical isolates of MRSA. The antimicrobial activity of the supernatant was also efficient against pathogenic fungi, such as a clinical isolate from a *Candida albicans* subspecies (Figure 1C). In contrast, no considerable activity was observed against Gram-negative strains (Table 1).

### 3.2. 16S rRNA Gene Sequencing and Phylogenetic Analysis

**Figure 2:**
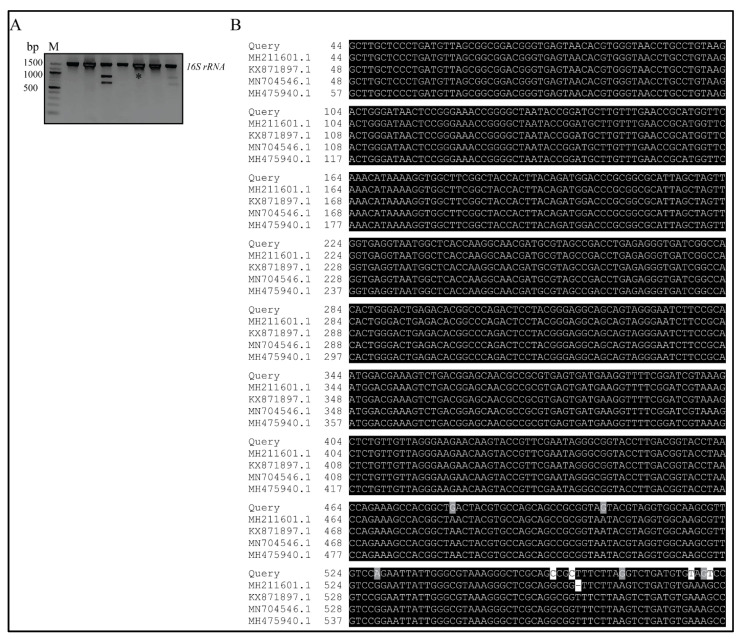
Molecular characterization and phylogenetic analysis of the antibiotic-producing strain BS-40. (**A**) Electrophoretic graph of PCR products on a 1.5% agarose gel stained with ethidium bromide for the *16S rRNA* gene among seven soil bacterial isolates; positive isolates yielded a single band of 1500 bp. * refers to BS-40. (**B**) Multiple sequence alignment of one category of the identified *16S rRNA* gene of isolate BS-40 (Query) with the related sequences of *16S rRNA* genes from bacterial species identified from a BLASTN search of GenBank that exhibited high homology to the queried sequence. The available sequences are aligned via ClustalW, and BOXSHADE was used to highlight the multiple alignment. Black shading indicates that the residue is identical to the column consensus; gray shading indicates that the residue is not identical but at least similar to the column consensus; and no shading indicates that the residue is neither identical nor similar to the consensus.

Molecular characterization of the antibiotic-producing *Bacillus sp.* was performed by PCR amplification of the 16S rRNA gene from strain BS-40 and resulted in a 1452 bp fragment (Figure 2A) that was sequenced to recognize the bacillus species it belongs to. After trimming ambiguous nucleotides using BioEdit software (v7.2.5.) [45], a sequence of 588 bp in length was deposited to the GenBank under Accession No. MZ198347. Absolute homology was detected upon alignment of the sequenced 16S rRNA of the isolated strain against a library of 16S rRNA of *Bacillus subtilis* subsp., including: *Bacillus subtilis* subsp. *spizizenii* strain NDS6 16S (GenBank Accession No. KX871897.1) with nucleotide identity ~99.0%), *Bacillus subtilis* subsp. *stercoris* strain EGI160 (GenBank Accession No. MN704546.1) with nucleotide identity ~98.0%, *Bacillus subtilis* strain JP2 (GenBank Accession No. MH475940.1) with nucleotide identity of ~98.0%, and *Bacillus subtilis* strain Khozestan2 (GenBank Accession No. MH211601.1) with nucleotide identity ~98% (Figure 2B). 

**Figure 3:**
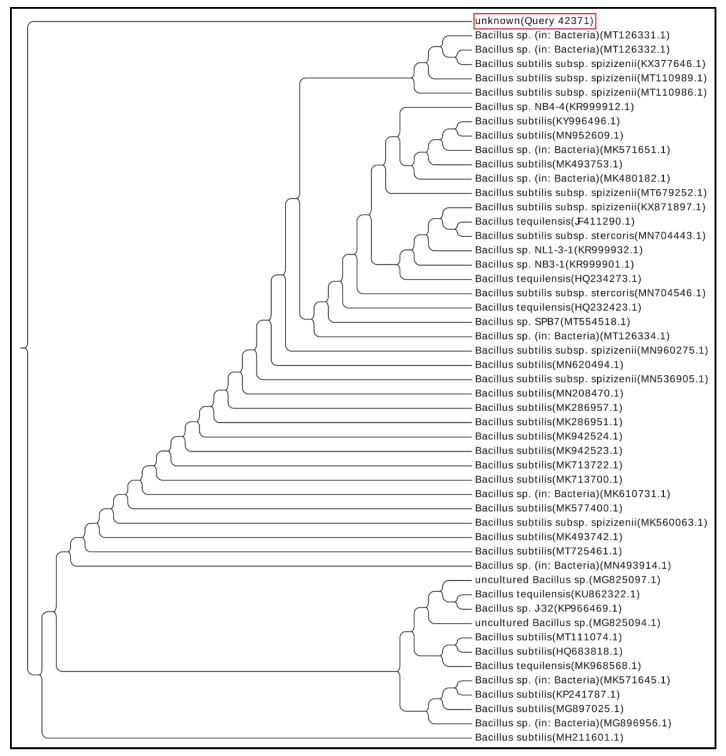
Phylogenetic tree based on the similarity of *16S rRNA* genes showing that the BS-40 strain (red outline) is closely related to a subspecies of *Bacillus subtilis*.

On the basis of a BLASTN search of GenBank, the phylogenetically closest matches to strain BS-40 were a closely related subspecies of *Bacillus subtilis*, according to the phylogenetic tree created from the 50 most related organisms to the queried 16S rRNA (Figure 3).

### 3.3. Optimization of the Antimicrobial Productivity

**Figure 4:**
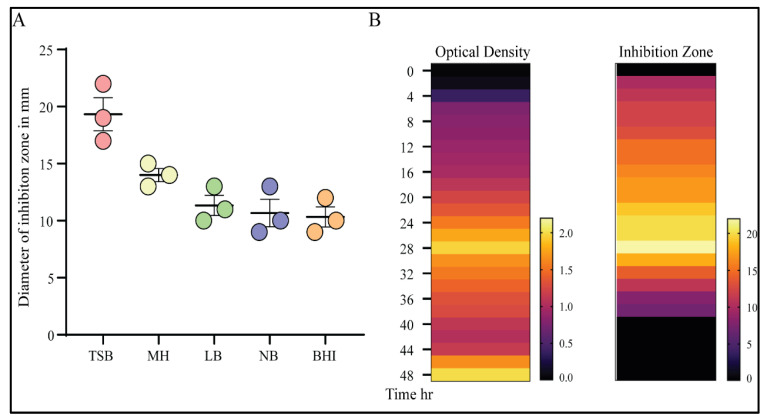
Kinetics of antibiotic production: (**A**) The bacterial isolate was grown in different media (TSB, MH, LB, NB, and BHI). Each supernatant was then filtered, anti-MRSA activity was assayed using the well diffusion method, and the diameter of the zone of inhibition was measured in millimeters after 48 h of incubation. Three independent biological replicates were performed. (**B**) Heat maps depicting kinetics of bacterial growth in TSB media and corresponding antibiotic productivity dictated by measuring the zone of inhibition over time for 48 h.

The antimicrobial productivity of the identified strain was tested in various media. The TSB medium enabled the maximum biomass accumulation and high antibacterial activity against MRSA-1 isolates compared with MH, LB, NB, or BHI broth media (Figure 4A). A variation in culture medium composition was previously shown to affect the production and/or secretion of the antimicrobial activity [46]. Following the analysis of the growth kinetics and antimicrobial activity of the supernatant, the antimicrobial agent was optimally produced at 37 °C after 24–28 h of growth (Figure 4B). Moreover, the antimicrobial activity of the supernatant was not related to an organic acid effect, as the measured pH of the culture medium was in the neutral range.

### 3.4. Effect of pH and Temperature on the Efficacy of the Antimicrobial Compound

The antimicrobial compound was stable at pH 7, but the activity was reduced to 77% by decreasing the pH from 6 to 2. Moreover, the activity was reduced to 72 and 66% at pH 8 and pH 9, respectively. The activity also sharply declined at pH 10. The antimicrobial molecule was thermostable at a wide range of temperatures up to 70 °C while preserving full antimicrobial activity. Activity decreased to 94% at a temperature of 80 °C (Table 4). The stability of this antibiotic as evidenced by maintenance of the antibacterial activity over a wide range of pH and temperature values suggests its usefulness in various industrial and veterinary applications.

### 3.5. Effect of Surfactant and Metal Salts

We tested the biocompatibility of the isolated antibiotic with different metal salts and surfactants. The antimicrobial activity was not affected by non-ionic surfactants at a final concentration of 1%. Moreover, the antibiotic was compatible with a wide range of metal ions (Table 5), which indicates a lack of antagonistic complexation between metal ions and the tested antibiotic.

### 3.6. Chemical Characterization and Qualitative Tests for Identification

**Figure 5:**
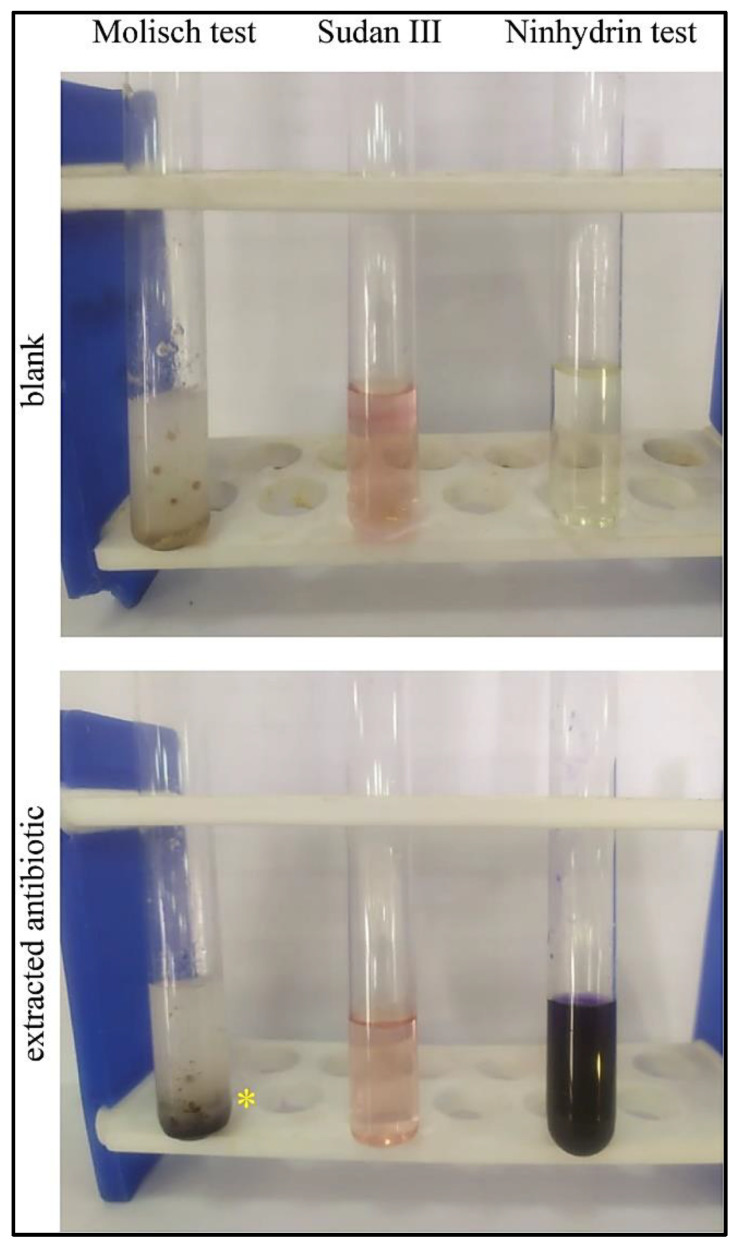
Chemical characterization of the antibiotic. The extracted antibiotic was tested for the presence of sugar residues using the Molisch test, for fatty acid or lipid residues using Sudan III, and for peptide residues using the ninhydrin reagent. A blank was tested for the same series of reagents as a negative control. The extracted antibiotic was positive in the Molisch test (violet ring indicated by *) and interacted positively with the ninhydrin reagent (development of purple color), whereas the antibiotic behaved the same as the blank when mixed with Sudan III.

After a series of traditional qualitative chemical tests to characterize the purified antimicrobial agent and verify its chemical composition, the purified antibiotic produced positive results when tested against a ninhydrin reagent, which indicates the presence of amino acids or peptide residues within the chemical structure [47]. Similarly, the purified antibiotic showed a positive reaction in the Molisch test, which indicates the presence of sugar or carbohydrate residues [48], whereas a negative result was observed upon treatment with Sudan III (lipid staining dye), which excludes the existence of lipid residues [49] or fatty acids in the structure of the purified antibiotic (Figure 5). Taken together, the purified antibiotic consists chemically of a glycone, mainly a sugar, and an aglycone part (non-sugar) of peptide nature.

### 3.7. Effect of Bacillus subtilis Extract on the % Area of Corneal Opacity

**Figure 6:**
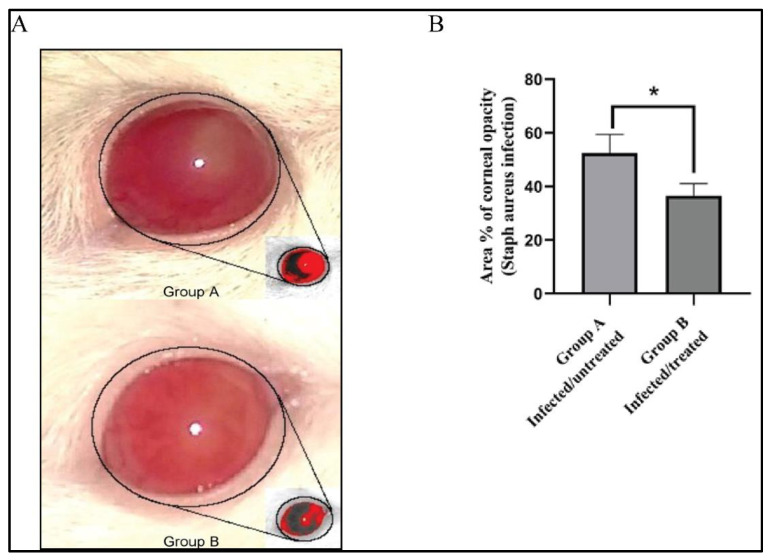
Effect of *Bacillus subtilis* extract on the % area of corneal opacity. (**A**) Untreated immunocompromised rats that were infected with MRSA (group A) and immunocompromised rats that were infected with MRSA and treated with *Bacillus subtilis* BS-40 extract (group B). The red color in the pictures defines the areas with focal lesions (arrows). By using ImageJ, the original images were converted to grayscale, then the opaque areas were segmented using thresholding followed by automatically measuring the thresholded area. (**B**) Quantification of the effect of *Bacillus subtilis* BS-40 extract on the % area of corneal opacity on untreated immunocompromised rats that were infected with MRSA (a) and immunocompromised rats that were infected with MRSA and treated with *Bacillus subtilis* BS-40 extract (b). * vs. group A (infected/untreated).

*Staphylococcus aureus* is one of the principal pathogenic agents in keratitis, which is a vision-threatening disease that causes endophthalmitis and vision loss [50]. We tested the in vivo activity of the newly isolated antibiotic against eye infections in rats. On the third day after infection, the infected eyes were photographed, and the images were processed using Image J 1.52i software (NIH, Bethesda, MD, USA). As shown in Figure 6A, the red color defines the areas of focal infection. As a measure of the clinical presentation of MRSA infection, the % area of opacity was calculated. Treatment of keratitis with the antimicrobial agent isolated from *Bacillus subtilis* considerably diminished the infection as evidenced by a significant decrease in the % area of opacity compared with the untreated group A (Figure 6B). 

## 4. Discussion

Finding new antibiotics and designing new antimicrobials to fight drug resistant pathogens represent a lifesaving strategy and a focus of microbiological research. The emergence of antibiotic-resistant pathogens directly threatens lives and represents a major challenge in the control of infectious diseases caused by these pathogens. MRSA is one of the most notorious and dangerous microorganisms that causes nosocomial infections. MRSA has the ability to readily acquire resistance to different types of antibiotics, which limits treatment success [7,51]. Therefore, there is an urgent need to discover effective antimicrobial compounds against MRSA.

In the present study, we screened for antibiotic-producing bacteria from soil samples. We discovered an isolate that exhibited antibacterial activity against MRSA. The bacterium was purified, identified phenotypically by microscopic examination and different biochemical tests (Figure 1A,B) and finally, the 16S rRNA gene was sequenced for molecular identification. This resulted in the identification of the isolate as *Bacillus subtilis* (strain BS-40) (Figure 2B,C and Figure 3). The antimicrobial agent isolated from this strain was further examined through a series of physical and chemical tests. The antimicrobial agent exhibited a narrow spectrum of action limited to Gram-positive bacteria (Table 2) and *Candida albicans*; however, it exhibited a promising antimicrobial effect against MRSA (Figure 1C). The antimicrobial agent showed high stability against elevated temperature and conserved 100% activity at temperatures up to 70 °C and up to 64% activity after autoclaving (Table 4). Monitoring the kinetics of bacterial growth and the corresponding antimicrobial activity of the purified supernatant, we found that the antimicrobial compound was maximally produced upon culturing the bacterial isolate in TSB media (Figure 4A), and the optimum yield was achieved upon growing the bacteria for 24–28 h at 37 °C (Figure 4B).

Some antibiotics function optimally at alkaline pH (e.g., aminoglycosides, fluoroquinolones, and macrolide), whereas others work maximally in acidic pH (e.g., tetracyclines and β-lactams) [52]. In this regard, the extracted antimicrobial agent in our study retained good activity (77%) in acidic conditions up to pH 2, whereas it lost its antimicrobial activity in the alkaline medium above pH 9 (Table 4). The good stability of this antibacterial agent after heat treatment and its activity over a wide pH range renders it useful for a variety of applications and industrial processes [7]. Certain categories of antibiotics are extensively inactivated by surfactants or metal ions that form sparingly soluble complexes and influence their bioavailability [53,54,55]. The antimicrobial activity of the purified antibiotic in our study was not affected by either the presence of surfactant or different metals as shown in Table 5.

Several *Bacillus* species produce peptide antibiotics that are encoded in their genomes and synthesized by either ribosomal or non-ribosomal mechanisms [6,56,57,58]. Glycopeptide antibiotics have a narrow spectrum of activity; however, they are considered the last option for the treatment of life-threatening infections caused by Gram-positive human pathogens, such as MRSA and *Clostridium difficile* [59,60]. The isolated antibiotic that we discovered has a glycopeptide structure as determined by chemical analysis. This compound interacted positively with the Molisch reagent, indicating the existence of a sugar or a carbohydrate residue in the chemical structure. It also interacted positively in the ninhydrin reagent test, indicating the presence of a peptide residue in the chemical structure. However, the existence of fatty acids or lipid residues was excluded, since no change in the color of Sudan III (lipid detecting reagent) was observed (Figure 5). Finally, the antibiotic showed promising antimicrobial activity when tested in vivo for eye infections caused by MRSA in rats (Figure 6).

## 5. Conclusions

In conclusion, the soil-isolated *Bacillus subtilis* strain (BS-40) contains a novel antimicrobial metabolite with promising biocidal activity against Gram-positive bacteria and *Candida albicans*. The antimicrobial compound displayed remarkable stability against extreme temperatures, pH levels, non-ionic surfactants, and some various metal ions. The antibiotic has a glycopeptide-like structure as evidenced by chemical tests for sugar, fatty acids, and peptide residues. Interestingly, the extracted antibiotic exhibited significant activity against MRSA in vitro and in vivo when tested against MRSA-induced keratitis in rats. The newly isolated antibiotic may represent an alternative to vancomycin to manage MRSA infections. Further studies should be performed on the extracted compound including spectroscopic analysis to identify its precise chemical structure, to optimize production, and to identify its structure–activity relationship.

## Figures and Tables

**Table 1:** List of primers with annealing temperatures and expected size of amplicons in this study.

Primer	Sequence (5′-3′)	Amplicon Size	Target	Annealing Temperature	Ref.
*mecA*-F	GTAGAAATGACTGAACGTCCGATAA	310 bp	*mecA* gene	52 °C	[31]
*mecA*-R	CCAATTCCACATTGTTCGGTCTAA				
*16S rRNA*-F	AAGTCGAACGGACACGCAT	1492 bp	*16S-rRNA*	52 °C	[32]
*16S rRNA*-R	TACGGATACCTTGTTACGACTT				

**Table 2:** Summary of the antimicrobial activity spectrum of the supernatant of *Bacillus sp*. using cut-well agar diffusion assay.

Isolate No.	Indicator Organisms	Media	Zone of Inhibition (Diameter in mm)
1	*Staphylococcus aureus* ATCC 29312	MH	17
2	*Staphylococcus epidermidis* *	MH	14
3	Methicillin resistant *Staphylococcus aureus* 1 *	MH	17
4	Methicillin resistant *Staphylococcus aureus* 2 *	MH	16
5	Methicillin resistant *Staphylococcus aureus* 3 *	MH	15
6	Methicillin resistant *Staphylococcus aureus* 4 *	MH	12
7	*Candida albicans* *	SDA	30
8	*Escherichia coli* *	MH	No zone
9	*Escherichia coli* ATCC 12435	MH	No zone
10	*Klebsiella pneumonia* *	MH	No zone
11	*Pseudomonas aeruginosa* *	MH	No zone
12	*Proteus mirabilis* *	MH	No zone

* indicates clinical strains.

**Table 3:** Treatment protocol.

Group	Ketamine/Xylazine	Methyl Prednisolone	MRSA Infection	*Bacillus subtilis* Extract
**Group A**	+	+	+	-
**Group B**	+	+	+	+

**Table 4:** Effects of pH and temperature on the antimicrobial activity.

Treatment	Antimicrobial Activity% of Control Value
**Untreated (Control)**	100 ± 4.26
**pH**	
2.0	77.203 ± 6.5 *
3.0	77.31 ± 5.34 *
4.0	77.42 ± 5,66 *
5.0	77.1 ± 6.43 *
6.0	77.22 ± 4.38 *
7.0	100.36 ± 5.32
8.0	72.41 ± 6.2 *
9.0	66.33 ± 5.87 *
10.0	0 *
11.0	0 *
12.0	0 *
**Temperature**	
40 °C	100 ± 3.23
50 °C	100.36 ± 6.32
60 °C	100.38 ± 4.7
70 °C	100.43 ± 5.32
80 °C	100.4 ± 6.47
90 °C	94.44 ± 5.2
100 °C	94.22 ± 4.29
Autoclaving 121 °C for 20 min	94.34 ± 4.82

* indicates statistically significant at *p* < 0.05.

**Table 5:** Effects of surfactants and metal salts on the antimicrobial activity.

Treatment	Antimicrobial Activity
**Surfactants**	
Sodium dodecyl sulfate (SDS)	+
Tween 80	+
**Metal Salts**	
FeSO_4_	+
MgSO_4_	+
AgNO_3_	+
ZnSO_4_	+
CdCl_2_	+
CuSO_4_	+
CaCl_2_	+
NiCl_2_	+

+ indicates the retention of activity.

## Data Availability

The data presented in this study are available on request from the corresponding author.
